# High loading of nanostructured ceramics in polymer composite thick films by aerosol deposition

**DOI:** 10.1186/1556-276X-7-92

**Published:** 2012-01-27

**Authors:** Hyung-Jun Kim, Song-Min Nam

**Affiliations:** 1 Department of Electronic Materials Engineering, Kwangwoon University, 447-1 Wolgye-dong, Nowon-gu, Seoul 139-701, South Korea

**Keywords:** aerosol deposition, Al_2_O_3_, polyimide, polymer composite, integrated substrate, high loading of ceramics, system-on-package

## Abstract

Low temperature fabrication of Al_2_O_3_-polyimide composite substrates was carried out by an aerosol deposition process using a mixture of Al_2_O_3 _and polyimide starting powders. The microstructures and dielectric properties of the composite thick films in relation to their Al_2_O_3 _contents were characterized by X-ray diffraction analysis. As a result, the crystallite size of α-Al_2_O_3 _calculated from Scherrer's formula was increased from 26 to 52 nm as the polyimide ratio in the starting powders increased from 4 to 12 vol.% due to the crushing of the Al_2_O_3 _powder being reduced by the shock-absorbing effect of the polyimide powder. The Al_2_O_3_-polyimide composite thick films showed a high loss tangent with a large frequency dependence when a mixed powder of 12 vol.% polyimide was used due to the nonuniform microstructure with a rough surface. The Al_2_O_3_-polyimide composite thick films showed uniform composite structures with a low loss tangent of less than 0.01 at 1 MHz and a high Al_2_O_3 _content of more than 75 vol.% when a mixed powder of 8 vol.% polyimide was used. Moreover, the Al_2_O_3_-polyimide composite thick films had extremely high Al_2_O_3 _contents of 95 vol.% and showed a dense microstructure close to that of the Al_2_O_3 _thick films when a mixed powder of 4 vol.% polyimide was used.

## Introduction

Electronic devices have recently undergone rapid progress in terms of their multifunctionality, speed, and miniaturization. These desired properties have produced many studies into the technology and integration of components on substrates, such as printed circuit boards [PCB], multi-chip modules, and system-in-a-package methodologies [1-4]. As a next generation electronic packaging technology, system-on-package integrates both the active components (digital integrated circuits [ICs], analog ICs, memory modules, and MEMS) and the embedded passive components (capacitors, resistors, and inductors) into a multilayer-integrated substrate and provides an improved miniaturization through three dimensional [3-D] lamination [5-7]. Moreover, the high-frequency properties of the components have grown in importance due to rising demands on wireless communications. However, conventional polymer-based PCB substrates are not suitable for high-frequency applications, such as embedded RF, since these applications require high quality factors [Qs] [8]. In comparison, ceramic substrates have high Qs, excellent thermal conductivity, and low coefficients of thermal expansion close to those of Si. However, the ceramics have some fundamentally weak characteristics, such as brittleness, poor plasticity, and a high processing temperature of over 1,000°C. The high processing temperature needed for ceramics is a critical problem that must be solved in order to achieve 3-D integration because the embedded metal transmission lines and polymer insulation films cannot tolerate high temperatures [9]. For this reason, many studies have been carried out regarding low temperature processes for ceramic-based substrates. Polymer composites are a candidate for low temperature fabrication technology, but it is difficult to increase the ceramic content, which offers superior dielectric and thermal properties at levels above 60 vol.% [10-12].

In order to overcome this problem, our research group has studied the aerosol deposition method [AD]; based on its room-temperature process [13,14], it can easily form composites in the submicron range using different kinds of materials, such as ceramics, polymers, or metals by simply mixing their starting powders [15-18]. In this study, we attempted to fabricate Al_2_O_3_-polyimide composite thick films with high Al_2_O_3 _contents of more than 60 vol.% and studied the characteristics of these composite thick films in relation to their contents of Al_2_O_3_.

## The experiment

The AD method is based on the principle of particle collision. A starting powder forms an aerosol in an aerosol chamber by mixing with the carrier gas controlled by a mass flow controller, and a vibration system under the aerosol chamber helps to generate the aerosol. The aerosol is transferred to a nozzle in the deposition chamber through a pipe line by a pressure difference generated by vacuum pumps. The aerosol is accelerated to a velocity of several hundred meters per second by the flow of the gas through a nozzle and then sprayed onto a substrate. In order to obtain uniform thick films, the substrate is continuously moved. Dense thick films are grown through the impact of the powder on the substrate in the deposition chamber at room temperature.

A commercial polyimide powder (BMI-5100, Daiwa Kasei IND, Wakayama, Japan) was milled to decrease the powder size by a planetary ball mill (Pulverisette 5, Fritsch, Idar-Oberstein, Germany) so that a polyimide starting powder with a 1-μm average diameter was obtained. We used α-Al_2_O_3 _powder with a 0.5-μm average diameter (99.4% purity, AL-160SG3, Showa-Denko K.K., Tokyo, Japan) as the ceramic starting powder. The Al_2_O_3 _powder was heated to 900°C for 2 h before deposition in order to improve its dielectric properties [15]. The Al_2_O_3 _powder was mixed with the polyimide powder at volume ratios of 4%, 8%, and 12% using the ball mill.

The Al_2_O_3_-polyimide composite thick films were deposited on Cu and glass substrates by AD at room temperature. Table 1 shows the deposition conditions. The microstructures of the composite thick films were examined by scanning electron microscopy [SEM] and transmission electron microscopy [TEM]. An X-ray diffraction [XRD] analysis was performed to confirm the existence of α-Al_2_O_3 _in the composite thick films and to examine the variations in crystallinity according to the changes in the mixing ratio. The crystallite size of the α-Al_2_O_3 _in the films was calculated using Scherrer's formula. The dielectric properties were measured from 1 kHz to 10 MHz using an impedance analyzer. In order to measure the dielectric properties of the deposited thick films, Au electrodes of 1.5 mm in diameter were sputtered onto the surface of the composite thick films. Finally, the Al_2_O_3 _content in the fabricated composite thick films was calculated from the relative permittivity using the Hashin-Shtrikman theory [19] and electrostatic simulations. Previous research showed that Al_2_O_3_-based polymer composite thick films fabricated by AD were well matched at the bottom limits of the Hashin-Shtrikman bounds with errors of less than 5% [16].

**Table 1:** The AD parameters for the Al_2_O_3_-polyimide composite thick films

Deposition conditions	
Starting powder	Ceramic: α-Al_2_O_3_ Polymer: polyimide
Substrate	Cu and glass
Carrier gas	He
Size of nozzle orifice	10 × 0.4 mm^2^
Scanning speed	1 mm/sec
Working pressure	6-8 Torr
Consumption of carrier gas	1-2 L/min
Distance between substrate and nozzle	10 mm
Deposition temperature	Room temperature
Deposition time	10-40 min
Deposition area	10 × 10 mm^2^

## Results and discussions

**Figure 1:**
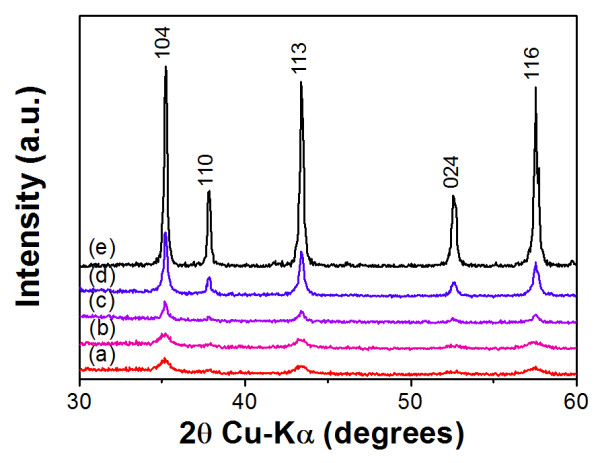
**X-ray diffraction patterns**. The X-ray diffraction patterns of (a) Al_2_O_3 _thick film, (b) Al_2_O_3_-polyimide composite film (4 vol.%), (c) Al_2_O_3_-polyimide composite film (8 vol.%), (d) Al_2_O_3_-polyimide composite film (12 vol.%), and (e) α-Al_2_O_3 _starting powder.

The Al_2_O_3_-polyimide composite thick films were deposited on Cu substrates using the mixed starting powders by AD at room temperature. The Al_2_O_3 _thick films and polyimide thick films were also fabricated to compare the crystallinity and dielectric properties of the films. Figure 1 shows the XRD patterns of deposited films with different mixing ratios of the Al_2_O_3 _starting powder. The α-Al_2_O_3 _phase of the Al_2_O_3 _starting powder could be confirmed in the deposited Al_2_O_3 _thick films as well as in all of the composite thick films. The diffraction pattern of the Al_2_O_3 _thick film showed peak broadening and decreased intensity in comparison with that of the Al_2_O_3 _starting powder as shown in Figure 1a. This result can be explained by the presence of nanocrystallites in the films, which were generated by particle crushing during the deposition [13]. In comparison, the peak patterns of the Al_2_O_3_-polyimide composite thick films became sharp and strong as the polyimide ratio in the starting powders increased.

**Figure 2:**
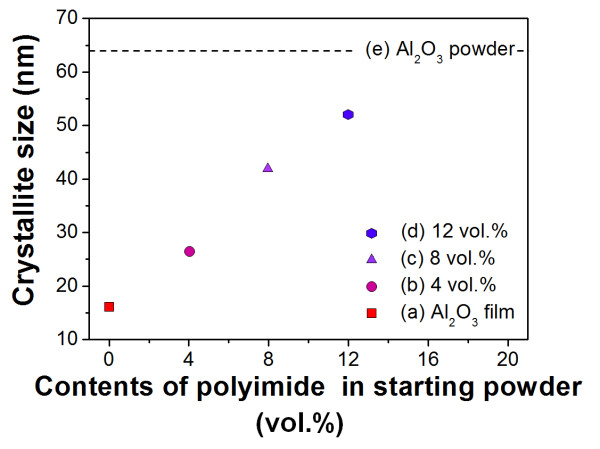
**Crystallite sizes**. The crystallite sizes of (a) Al_2_O_3 _thick film, (b) Al_2_O_3_-polyimide composite film (4 vol.%), (c) Al_2_O_3_-polyimide composite film (8 vol.%), (d) Al_2_O_3_-polyimide composite film (12 vol.%), and (e) α-Al_2_O_3 _starting powder as calculated using Scherrer's formula.

Also, the crystallite size of α-Al_2_O_3 _calculated from Scherrer's formula was increased from 26 to 52 nm as the polyimide ratio in the starting powders increased from 4 to 12 vol.% as shown in Figure 2. This result can be attributed to the decrease of the crystallite size after deposition due to the crushing of the starting powder being reduced by the shock-absorbing effect of the polyimide.

**Figure 3:**
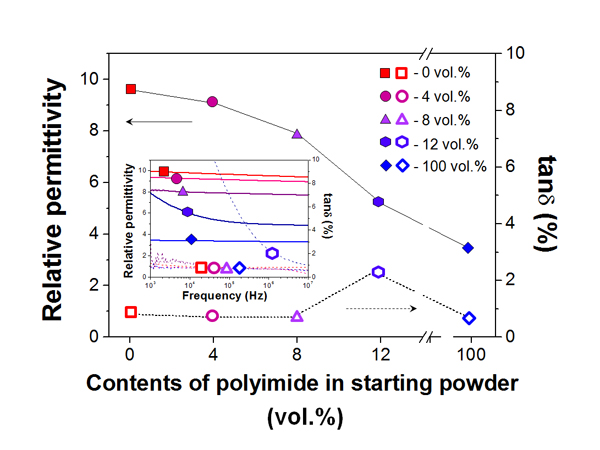
**The dielectric properties of the deposited films in relation to the contents of polyimide**.

Figure 3 shows the dielectric properties of the films fabricated by AD. The relative permittivity of the Al_2_O_3_-polyimide composite thick films decreased as the polyimide ratio in the starting powders increased. For the loss tangent, all composite thick films showed a low loss tangent of less than 1%, except for the composite thick film that was made using the starting powder of 12 vol.% polyimide. The Al_2_O_3_-polyimide composite thick film made using the starting powder of 12 vol.% polyimide showed a high loss tangent of close to 3% and a large frequency dependence. In order to confirm the cause of the increased loss tangent in this film, the microstructures of the films were analyzed through SEM observations.

**Figure 4:**
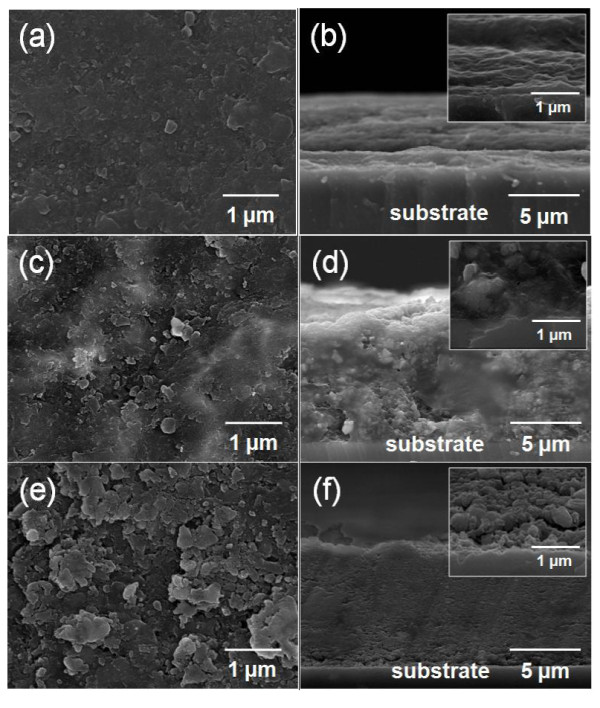
**Surface and cross-sectional SEM images**. The surface and cross-sectional SEM images of the Al_2_O_3_-polyimide composite thick films with different mixing ratios for the polyimide in the starting powder: **(a) **and **(b) **show the 4 vol.% composite, **(c) **and **(d) **show the 8 vol.% composite, and **(e) **and **(f) **show the 12 vol.% composite.

Figure 4 shows the microstructures of the Al_2_O_3_-polyimide composite thick films fabricated by AD. The surface roughness increased as the polyimide ratio increased in the starting powder as shown in Figure 4a,c,e. The cross-sectional SEM observations showed more clearly the structural changes in the Al_2_O_3_-polyimide composite thick films caused by the increase of the polyimide content. The Al_2_O_3_-polyimide composite thick film made using the starting powder of 4 vol.% polyimide showed a dense microstructure close to that of the Al_2_O_3 _thick films. In the case of the composite film made by using the starting powder of 8 vol.% polyimide, there were submicron Al_2_O_3 _particles with dense microstructure in the composite film. In the case of the composite film made using the starting powder of 12 vol.% polyimide, however, the film density was deteriorated and the porosity was increased due to the excessive amount of polyimide. It was estimated that the increased loss tangent in the composite thick films made using the starting powder of 12 vol.% polyimide was caused by the rough surface and increased porosity of these films. The increased surface area and open pores could have caused the increase in the loss tangent by facilitating the absorption of moisture [20].

**Figure 5:**
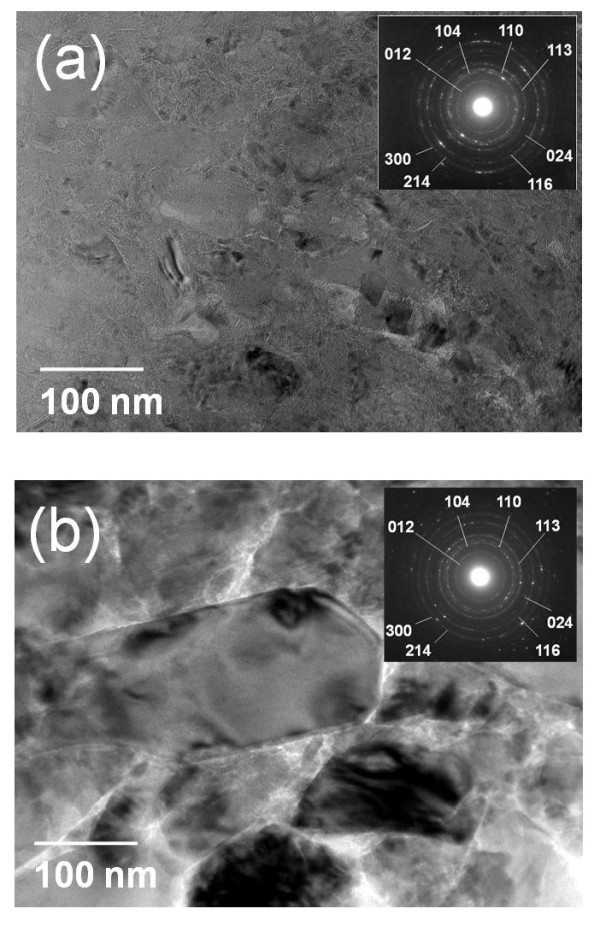
**TEM images and the selected area electron diffraction [SAED] patterns**. The TEM images and the SAED patterns of the AD thick films: **(a) **Al_2_O_3 _thick film and **(b) **Al_2_O_3_-polyimide composite thick film (starting powder, 8 vol.%).

The TEM images of the Al_2_O_3_-polyimide composite thick films showed the differences between these films and the Al_2_O_3 _thick films more clearly. Figure 5 shows the TEM images of the Al_2_O_3 _thick film and the Al_2_O_3_-polyimide composite thick film made using the starting powder of 8 vol.% polyimide. As shown in Figure 5a, the microstructure of the Al_2_O_3 _thick film showed a polycrystalline structure consisting of nanocrystallites with sizes between 5 and 20 nm. It has been suggested that the nanocrystallites are formed by the fracturing of the Al_2_O_3 _starting powder during the film growth. In comparison, the Al_2_O_3_-polyimide composite thick films included large Al_2_O_3 _crystallites that are greater in size than 100 nm as shown in Figure 5b. It is thought that the relatively soft polyimide powders prevent the crushing of the Al_2_O_3 _particles when the Al_2_O_3 _particles collide with the substrate.

**Figure 6:**
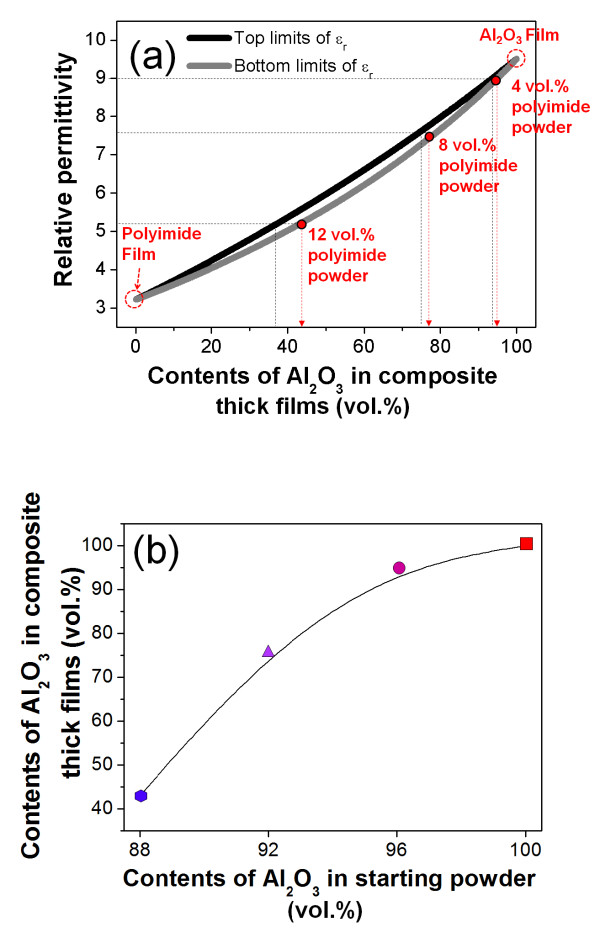
**Calculation of the contents of the Al_2_O_3 _in the composite thick films**. The calculation of the contents of the Al_2_O_3 _in the composite thick films: **(a) **the Hashin-Shtrikman bounds of contents of Al_2_O_3 _in composite thick films as a function of measured relative permittivity and **(b) **the calculated contents of Al_2_O_3 _according to the contents of Al_2_O_3 _in the starting powders.

Finally, the Al_2_O_3 _content in the composite thick films was calculated from the relative permittivity using the Hashin-Shtrikman bounds and the electrostatic simulation. As a result, the possible range of the Al_2_O_3 _volume fraction in the Al_2_O_3_-polyimide composite thick films can be calculated as shown in Figure 6a. In our previous research, the dielectric properties of the Al_2_O_3_-based composite thick films were close to the bottom limits of the Hashin-Shtrikman bounds [16]. As a result, the relationship between the Al_2_O_3 _volume fractions in the Al_2_O_3_-polyimide composite thick films and the Al_2_O_3 _volume fractions in the starting powders was obtained from the bottom limits of the Hashin-Shtrikman bounds as shown in Figure 6b. The starting powder of 4 vol.% polyimide could achieve the highest Al_2_O_3 _content in the composites of close to 95 vol.%. However, we did not expect any relief of brittleness due to the dense microstructure of almost the Al_2_O_3 _thick films. Except for the above result, the Al_2_O_3_-polyimide composite thick films showed a high Al_2_O_3 _content of close to 75 vol.% with a uniform composite structure when the starting powder of 8 vol.% polyimide was used. As the result of this study, we could confirm the structural variations of the composite films according to the polyimide ratio and the possibility of AD as a solution for the high loading of ceramics in polymer composites.

## Conclusion

The Al_2_O_3_-polyimide composite thick films were deposited on Cu substrates by AD using mixed starting powders at room temperature. The crystallite size of α-Al_2_O_3 _in the composite thick films increased from 26 to 52 nm as the polyimide ratio in the mixed starting powders increased from 4 to 12 vol.%. The Al_2_O_3 _content was close to 95 vol.% when the mixed powder of 4 vol.% polyimide is used; however, the microstructure was close to that of the Al_2_O_3 _films. In the case of the mixed powder of 12 vol.% polyimide, the composite thick film showed a high loss tangent of close to 0.03 at 1 MHz and a large frequency dependence with a nonuniform microstructure. The Al_2_O_3_-polyimide composite thick films made using a mixed powder of 8 vol.% polyimide showed a uniform composite structure with a low loss tangent of less than 0.01 at 1 MHz and a high Al_2_O_3 _content of more than 75 vol.%.

## Competing interests

The authors declare that they have no competing interests.

## Authors' contributions

HJK carried out the aerosol-deposited sample fabrication, measurements, and interpretation of the results. SMN initiated the idea of working on the present topic and analyzed all experiments as a corresponding author. All authors read and approved the final manuscript.

## References

[B1] Shahparnia S Ramahi OM A simple and effective model for electromagnetic bandgap structures embedded in printed circuit boards IEEE Trans Electromagn Compat 2004 46 1 580 587 10.1109/TEMC.2004.837671

[B2] Laroussi R Costache GI Finite- element method applied to EMC problems IEEE Trans Elect Comp 1993 35 2 178 184 10.1109/15.229423

[B3] Roozeboom F Kemmeren ALAM Verhoeven JFC Heuvel FC Klootwijk J Kretschman H Fric T Grunsven ECE Bardy S Bunel C Chevrie D LeCornec F Ledain S Murray F Philippe P More than Moore: towards passive and system-in-package integration Thin Solid Films 2006 504 391 396 10.1016/j.tsf.2005.09.103

[B4] Gerlach P Linder C Becks KH Multi chip modules technologies Nucl Instr and Meth Phys Res A 2001 473 102 106 10.1016/S0168-9002(01)01128-7

[B5] Drevon C RF Packaging for space applications: from micropackage to SOP - "system on a package" Microelectron Reliab 2001 41 1649 1656 10.1016/S0026-2714(01)00168-8

[B6] Tummala RR Raj PM Atmur S Bansal S Banerji S Liu F Bhattachatya S Sundaram V Shinotani KI White G fundamental limits of organic packages and boards and the need for novel ceramic boards for next generation electronic packaging J Electroceram 2004 13 417 422 10.1007/s10832-004-5135-6

[B7] Tummala RR Swaminathan M Tentzeris MM Laskar J Chang GK Sitaraman S Keezer D Guidotti D Huang Z Lim K Wan L Bhattacharya SK Sundaram V Liu F Raj PM The SOP for miniaturized, mixed-signal computing, communication, and consumer systems of the next decade IEEE Trans Adv Packag 2004 27 2 250 267 10.1109/TADVP.2004.830353

[B8] Lee HJ Requirements of substrate materials for SOP (system on package) Ceramist 2005 8 3 25 30

[B9] Imanaka Y Takenouchi M Akedo J Ceramic dielectric film for microwave filter deposited at room temperature J Cryst Growth 2005 275 e1313 e1319 10.1016/j.jcrysgro.2004.11.107

[B10] Rao Y Ogitani S Kohl P Wong CP Novel polymer-ceramic nanocomposite based on high dielectric constant epoxy formula for embedded capacitor application J Appl Polym Sci 2002 83 5 1084 1090 10.1002/app.10082

[B11] Li H Liu G Liu B Chen W Chen S Dielectric properties of polyimide/Al_2_O_3 _hybrids synthesized by in-situ polymerization Mat Lett 2007 61 1507 1511 10.1016/j.matlet.2006.07.063

[B12] Kuo DH Chang CC Su TY Wang WK Lin BY Dielectric properties of three ceramic/epoxy composites Mater Chem Phys 2004 85 201 206 10.1016/j.matchemphys.2004.01.003

[B13] Akedo J Room temperature impact consolidation (RTIC) of fine ceramic powder by aerosol deposition method and applications to microdevices J Therm Spray Techn 2008 17 2 181 198 10.1007/s11666-008-9163-7

[B14] Nam SM Mori N Kakemoto H Wada S Akedo J Tsurumi T Alumina thick films as integral substrates using aerosol deposition method Jpn J Appl Phys 2004 43 5414 5418 12 10.1143/JJAP.43.5414

[B15] Kim HJ Nam SM Effects of heat treatment on the dielectric properties of aerosol-deposited Al_2_O_3_-polyimide composite thick films for room-temperature fabrication J Ceram Process Res 2009 10 6 817 822

[B16] Kim HJ Kim YH Nam SM Calculation of Al_2_O_3 _Contents in Al_2_O_3_-PTFE composite thick films fabricated by using the aerosol deposition J Korean Phys Soc 2010 57 4 1086 1091 10.3938/jkps.57.1086

[B17] Choi JJ Hahn BD Ryu JH Yoon WH Lee BK Park DS Preparation and characterization of piezoelectric ceramic-polymer composite thick films by aerosol deposition for sensor application Sens Act A 2009 153 89 95 10.1016/j.sna.2009.04.025

[B18] Han G Ryu JH Yoon WH Choi JJ Hahn BD Park DS Effect of film thickness on the piezoelectric properties of lead zirconate titanate thick films fabricated by aerosol deposition J Am Ceram Soc 2011 94 5 1509 1513 S 10.1111/j.1551-2916.2010.04276.x

[B19] Salvatore T Random Heterogeneous Materials: Microstructure and Macroscopic Properties 2005 New York: Springer-Verlag Press

[B20] Olhoeft GR Touloukian YS, Judd WR, Roy RF Electrical properties of rocks Physical Properties of Rocks and Minerals 1981 II-2 New York: McGraw-Hill 257 329

